# Efficient DNA Sampling in Burglary Investigations

**DOI:** 10.3390/genes13010026

**Published:** 2021-12-23

**Authors:** Colin Charles Tièche, Markus Dubach, Martin Zieger

**Affiliations:** 1 Forensic Molecular Biology Department, Institute of Forensic Medicine, University of Bern, Murtenstrasse 26, 3008 Bern, Switzerland; colin.tieche@irm.unibe.ch; 2 Bern Cantonal Police, Nordring 30, 3001 Bern, Switzerland; pmad@police.be.ch

**Keywords:** burglary, CODIS, touch DNA, forensic genetics, crime scene, authorized, sampling

## Abstract

In terms of crime scene investigations by means of forensic DNA-analyses, burglaries are the number one mass crime in Switzerland. Around one third of the DNA trace profiles registered in the Swiss DNA database are related to burglaries. However, during the collection of potential DNA traces within someone’s residence after a burglary, it is not known whether the sampled DNA originated from the perpetrator or from an inhabitant of said home. Because of the high incidence of burglaries, crime scene investigators usually do not collect reference samples from all the residents for economical and administrative reasons. Therefore, the presumably high probability that a DNA profile belonging to a person authorized to be at the crime scene ends up being sent to a DNA database for comparison, has to be taken into account. To our knowledge, no investigation has been made to evaluate the percentage of these non-perpetrator profiles straying into DNA databases. To shed light on this question, we collected reference samples from residents who had been victims of recent burglaries in their private homes. By comparing the profiles established from these reference samples with the profiles generated from trace DNA, we can show that the majority of the DNA samples collected in burglary investigations belong to the residents. Despite the limited number of cases included in the study, presumably due to a crime decline caused by the pandemic, we further show that trace DNA collection in the vicinity of the break and entry area, in particular window and door glasses, is most promising for sampling perpetrator instead of inhabitant DNA.

## 1. Introduction

Burglaries, usually coming along as a mixture of property damage, trespassing, and theft, are a severe type of property crime, the impact being increased by the feeling of insecurity left among the victims. In Switzerland and probably many other countries, burglaries constitute the number one volume crime investigated by means of forensic genetics. On one hand, it is a frequent type of property crime with 24,010 registered cases in Switzerland in 2020 (about 9% of all registered offences against property) [1] and on the other hand, burglaries are particularly suited for the sampling of DNA traces, compared to other volume crimes such as fraud. In the department for Forensic Molecular Biology of the Institute of Forensic Medicine, in which the present study was conducted, “touch” DNA traces from burglaries and burglary attempts make up for more than 50% of the overall number of traces analyzed.

Given the time spent within a household, one can expect that the majority of the DNA traces left on site actually originated from the respective inhabitants. However, despite the large number of cases, we rarely obtain reference samples from inhabitants to avoid having their profiles established from DNA traces sampled at the crime scene sent to the Swiss DNA database for comparison via CODIS. Assuming an average of 2–3 persons per household or even more for workspaces, when taking burglaries in business environments into account, routine sampling of reference swabs from all residents would cause enormous financial and administrative costs.

However, ignoring the fact that a large number of resident profiles end up registered in CODIS as traces profiles creates a couple of problems: (a) The police run the risk of spoiling investigations through the presentation of inadmissible evidence. The following real case might serve as an example to illustrate this particular problem: Two persons, a man and a woman living together, were assaulted by three men. From the jacket of the woman, a DNA mixture containing a female and a male component was recovered and sent to CODIS. The female component belonged to the female victim, as inferred by crosschecking all samples in the same case. The male component, however, resulted in profile matches with DNA traces from two burglaries. The police therefore started investigations against the male victim of the assault. The Bern Cantonal High Court, however, prohibited all the evidence gathered in this case from being used, since the investigation was launched based on inadmissible evidence given by two random and case unrelated DNA matches that could have been avoided by taking reference samples [2]. The verdict prevented the assailants from being brought to justice and the affaire became a cold case. (b) A lack of prior exclusion might prevent crime victims from contacting the police. The risk of being associated with a past crime or a juvenile sin, e.g., illegal graffiti spraying, might discourage people from contacting the police, once they have become victims themselves. In Switzerland, trace DNA sampling is legitimate in cases of property damage having caused more than 10,000 CHF of pecuniary damage, a sum that is easily reached through graffiti spraying. We can imagine a 16-year-old graffiti sprayer, who becomes a victim in a more serious crime and who refrains from contacting the police, because he fears that through incidental DNA matches, his past or present spraying activities might be revealed to the police. (c) The database becomes inflated. DNA trace profiles caused by residents will normally not generate hits in forensic databases, at least not with profiles of convicted individuals. Therefore, the traces will remain in the database for years and create candidate matches with newly added suspect profiles or trace profiles from other cases. DNA mixture profiles frequently lead to candidate matches that have to be verified by the submitting lab. The lab than decides whether the “candidate match” is considered as a true match (“HIT”), or not. For mixture profiles, most of the verifications result in “NO HIT” verdicts. The control of those candidate matches is time consuming and constitutes a possible source of error, justifying a cleanup of the database from profiles from crime-authorized people.

From controlled studies on DNA transfer and persistence [3,4,5,6], we expect a high number of the DNA trace profiles generated in burglary investigations to be from the inhabitants. However, no figures are available on how many of the profiles in real case scenarios stem from residents. To get an impression of those figures, we focused on burglaries in private households, because access is more restricted than in stores or other business environments. We collected the reference samples from the inhabitants and compared them to the DNA profiles generated from the crime scene traces before sending them for comparison into the Swiss DNA database.

## 2. Materials and Methods

### 2.1. Sampling

Sampling for crime scene traces in burglaries was performed by the Bern Cantonal Police in accordance with their usual practice. The police officers individually decided ad hoc at the scene where DNA from the offender could be expected and took samples accordingly. Reference samples from residents of private households were collected between November 2020 and April 2021. Residents present during the crime scene investigation were asked for reference samples. Consenting residents were handed out a written agreement form with explanations concerning the procedure, the modalities for the usage of their DNA profiles and information on why prior exclusion of their profiles would be in their favor. The police officer further registered the number of persons living in the household and whether there had been any visitors at the house within the last 7 days prior to the burglary. For all inhabitants not present at the apartment or house during the crime scene sampling, the police officer left a set of documents, buccal swabs, and a prepaid reply envelope on-site, so the absent inhabitants could send their reference swabs directly to the DNA laboratory by mail. All samples were given a pseudonymized family role (such as “father” or “man”) under which they were subsequently processed in the DNA laboratory. Buccal swabs were collected with Sarstedt Forensic Swab L. (Sarstedt AG, Nümbrecht, Germany), crime scene traces were collected using the forensiX Cardboard Evidence Collection Kit (Art. Nr. 9021040; Prionics, Schlieren, Switzerland). One or two partially moistened swabs were used for the sampling of one trace.

### 2.2. Analysis

DNA from buccal swabs was extracted by Chelex 100 (Bio-Rad Laboratories, Hercules, CA, USA). Samples were placed in 1500 µL of 20% Chelex 100, put for two times 30 s at 5900 rpm on a Precellys^®^24 homogenizer (Bertin instruments, Montigny-le-Bretonneux, France), incubated at 100 °C for 10 min and subsequently centrifuged at 13,000 rpm on an Eppendorf Minispin centrifuge (Eppendorf, Hamburg, Germany). DNA extracted from buccal swabs was quantified using a Qubit fluorometer (Thermo Fisher, Waltham, MA, USA). DNA profiles were established in 12.5 µL reaction volume by multiplex-PCR using the AmpFlSTR^®^ NGM Select Express^TM^ (Thermo Fisher, Waltham, MA, USA) and PowerPlex^®^ ESI17 Fast kits (Promega, Madison, WI, USA). DNA from trace samples was extracted with the AutoMateExpress^TM^ device and the PrepFiler Express^TM^ Kit (both Thermo Fisher, Waltham, MA, USA), with an elution volume of 50 µL, representing our standard lab procedure for swabs from touched surfaces [7]. DNA was quantified by Real-Time-PCR (qPCR) using the Quantifiler^®^ HP Kit from Thermo Fisher on a 7500 RT PCR System (Thermo Fisher, Waltham, MA, USA). DNA profiles were established by multiplex-PCR using the AmpFlSTR^®^ NGM Select^TM^ and NGMDetect^TM^ Kits (Thermo Fisher, Waltham, MA, USA) in a total reaction volume of 25 µL (at least two independent amplifications). A maximum of 0.5 ng DNA was amplified per reaction, using the maximum sample volume of 10 µL for samples with DNA-concentrations below 50 pg/µL. In line with our standard operating procedures for casework, all samples with a DNA concentration below 20 pg/µL were amplified with 32 instead of 30 PCR cycles. Capillary electrophoresis was run either on a 3130 xl or on a 3500 xl genetic analyzer (Thermo Fisher, Waltham, MA, USA). Signal interpretation was performed with Genemapper ID-X, v1.4 (Thermo Fisher, Waltham, MA, USA). All peaks above 50 rfu (3130 xl) and 100 rfu (3500 xl) were considered as true alleles.

### 2.3. Criteria for a Submission to Swiss CODIS Database

The Swiss DNA database uses the CODIS (combined DNA index system) software v7.0. Switzerland has defined by law the 16 loci included in the AmpFlSTR^®^ NGMSelect/NGMDetect and PowerPlex^®^ ESI17/ESX17 kits as database core loci [8]. More or less all offences leading to ordinary criminal proceedings are recordable, except misdemeanors penalized with a fine. Familial searches are permitted for all offences penalized with a prison sentence of more than three years and can be ordered by the public prosecutor. If reference samples are collected, the generated profiles are used only for the exclusion of people with lawful right of access to the scene. Reference profiles are not registered in CODIS. Profile exclusion is performed locally in the laboratory, before sending the trace profile to CODIS. The entry criteria for a regular database search in Switzerland are a minimum of 6 loci for single or major component profiles and 8 loci for two person mixtures. The sex locus Amelogenin can be entered, but is not searched for in CODIS. Apparent major components are selected, if no two-person mixture can be clearly distinguished. A submission was considered when the lab internal quality conditions were met, i.e., a minimum ratio of 3:1 for major to minor component and heterozygote peak balance of at least 60%. Profiles that did not fulfill our lab or the search criteria for CODIS, although they could possibly still be interpreted, e.g., by probabilistic genotyping [9,10], were not investigated any further. It is important to note that our study targets the investigative phase of burglary investigations and not the evaluative phase [11].

## 3. Results

### 3.1. Samples Obtained

We had a bad timing for our study, since we registered in the relevant timeframe between November 2020 and April 2021 an unusual low number of burglaries in private households. This was presumably due to the COVID-19 pandemic. As part of the measures taken in the fight against the pandemic, e.g., restrictions in cross-border traffic or the obligation of working in the home office, people spent more time at home. In 2020, we temporarily observed a workload drop of about 80%. From November to April, our lab registered 226 burglaries in private housings; attempts and sneak-in thefts not included. In 55 of those cases, so approximately one-quarter, we obtained the reference samples from the inhabitants. We analyzed 144 trace samples in total, what corresponds to 2.6 traces per burglary. We obtained a total of 118 references samples (50 women, 47 men, and 21 children), so in average about 2.14 reference samples per case, corresponding well with the average household size in Switzerland of 2.2 persons [12].

### 3.2. Sample Categories

**Figure 1:**
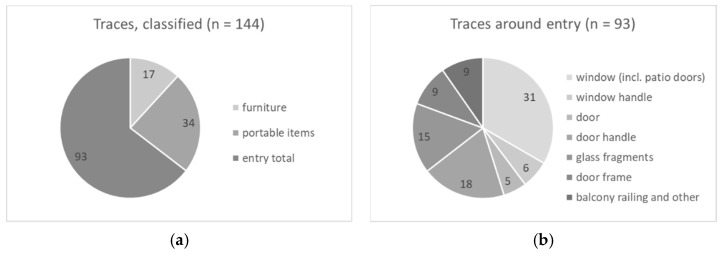
(**a**) All samples classified in three categories; (**b**) samples from entry divided in several sub-classes.

Samples were classified into three categories: (a) furniture, e.g., handles from cupboards, pieces of furniture that had been moved by the offenders; (b) portable items, e.g., keys or jewelry; and (c) samples taken around the entry of the offender, e.g., door or window. The majority, about 65%, of the samples were taken in the vicinity of the entry (Figure 1a). The samples around the entry were further divided into subcategories (Figure 1b). Half of them (*n* = 46) were collected from window glass or glass fragments.

### 3.3. Typing Success

**Figure 2:**
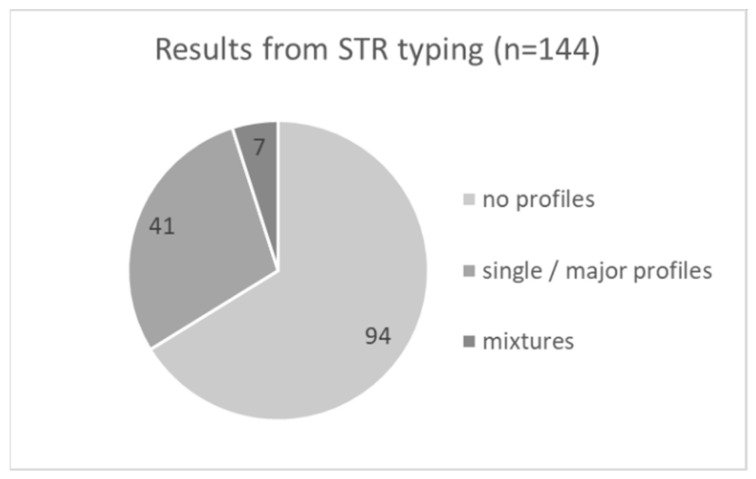
The STR typing results for all 144 crime scene samples summarized. The category “no profiles” includes all profiles not fulfilling the CODIS entry criteria.

We were able to establish DNA profiles suitable for submission to CODIS from 48 traces, what corresponds to an overall success rate of 33% (Figure 2).

**Figure 3:**
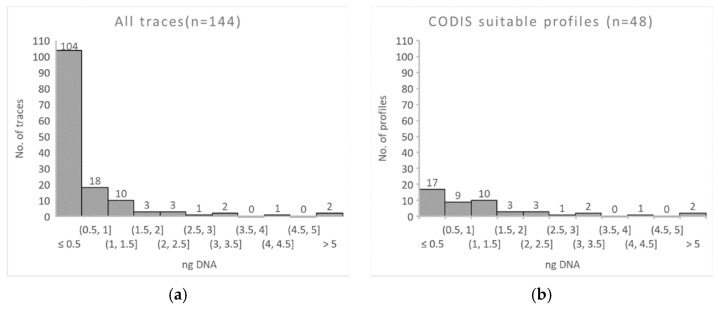
Histograms of the distributions of DNA amounts in ng obtained from: (**a**) all samples and (**b**) samples for which we were able to establish CODIS-suitable DNA profiles.

Figure 3 shows the distribution of the total amount of DNA extracted from each sample of all 144 samples (Figure 3a) and of the 48 samples that resulted in CODIS-suitable profiles (Figure 3b). We were able to establish DNA profiles for database entry for all traces with a total DNA amount of more than 1 ng. Around 85% of the traces with less than 0.5 ng total DNA did not result in CODIS-suitable profiles.

**Figure 4:**
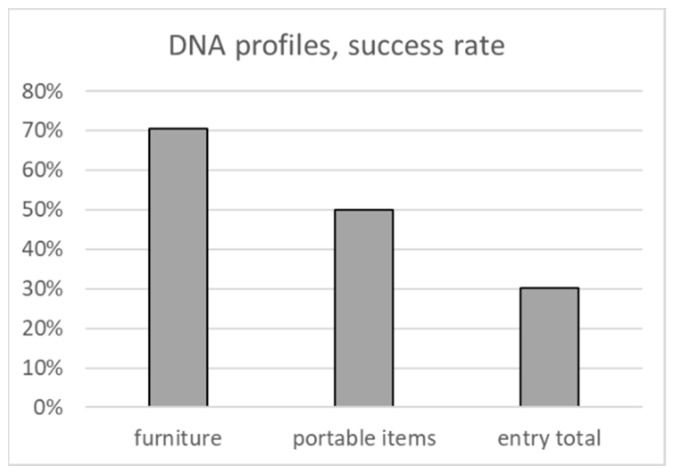
Fraction of samples of the respective class that resulted in CODIS-suitable DNA profiles.

The DNA typing success rate of samples taken from furniture and portable items reaches 70% and 50%, respectively, whereas only about 30% of traces taken around the entry resulted in CODIS-suitable profiles (Figure 4).

### 3.4. Comparison with Reference Samples

**Figure 5:**
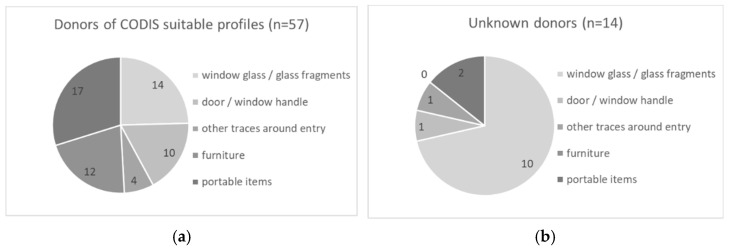
(**a**) Donors of the 48 CODIS-suitable profiles, according to trace class. (**b**) Unknown donors according to trace class. There were 0 unknown donors on furniture.

The 48 CODIS-suitable profiles include interpretable components from 57 DNA contributors. We obtained 41 single or major component profiles and 7 two-person mixtures. Two of the major component profiles showed a reproducible (at least three PCR amplifications conducted) minor component with more than five but less than eight alleles. Therefore, those three minor components are not suitable for CODIS as a mixture, but would be sufficient according to the Swiss CODIS criteria for interpretation, the reason why we included them in our interpretation. Of those 57 trace donors, 17 were detected on portable items, 12 on furniture and 28 on samples around the entry, half of which were from windows glass (Figure 5a). Of those 57 trace contributors, 43 (75%) were identified as crime scene authorized persons, i.e., inhabitants.

**Figure 6:**
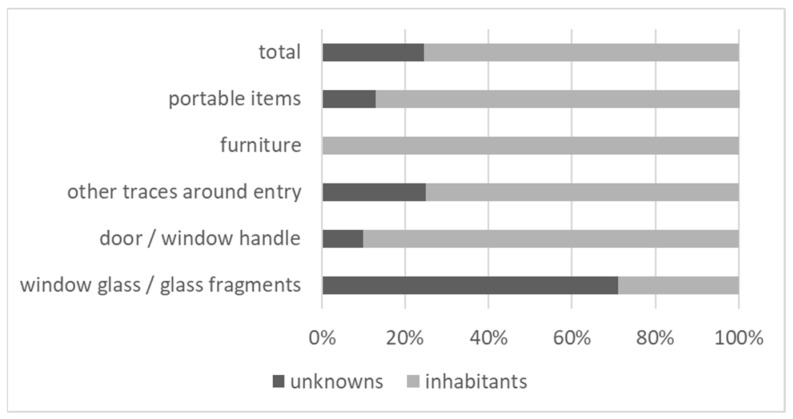
Ratios between unknown contributors and inhabitants detected on the different types of traces.

If we have a closer look on the different sample categories, we can see that traces from window glass clearly outperform the rest of the samples (Figure 5b). Whereas 71% of the interpretable traces detected on window glass and glass fragments are from unknown contributors, and therefore from possible suspects, less than 10% of the contributors from all other traces were from unknown individuals. The ratio of unknown vs. known contributors is depicted in Figure 6.

## 4. Discussion

The 33% success rate for DNA typing of contact (“touch”) traces in burglaries are in line with the approximate 25% overall typing efficiency we have observed in our lab in recent years. It also corresponds with the success rate from casework published by another Swiss laboratory, therefore operating within the same national and regulatory framework [13]. Their average success rate for “touch” DNA traces of 26% is slightly lower than the one for the samples from our study, but we have to take into account the higher sensitivity of the multiplex kits nowadays, since the study from 2008 used AmpFlSTR^®^ SGM Plus^TM^ and we used the more sensitive NGMSElect^TM^ and NGMDetect^TM^ kits.

Around 70% of the profiles established from window glass and glass fragments were from unknown individuals. A low level of background DNA on windows has been reported in a previous study, which suggests windows as premium choice for DNA sampling in burglary cases. However, no numbers were presented by the authors of this study from 2008 [14]. The results from our study confirm that windows have a comparatively low background of resident DNA and are therefore most suited for DNA trace analysis. We can see that the police officers of the Bern Cantonal Police are already focusing on the most promising sampling spots, since 32% of the traces we received were taken from windows or glass fragments.

Even though the data shown in Figure 6 seem to be very strong in favor of the sampling of window glass, we have to keep one limitation in mind, concerning the pre-selection effectuated by the police officer handling the case. This pre-selection is twofold: First, the officer will select the traces appearing most promising to him or her at the crime scene. Second, he or she will make a subsequent selection as to which samples to send for analysis to the DNA laboratory. If the analysis of the first samples sent to the lab do not result in interpretable DNA profiles, the case-managing officer might decide to send additional samples for analysis. Therefore, we encounter some risk of bias if samples from window glass are prioritized and no other samples are analyzed in the case. If we assume that the unknown profiles we can recover are maybe from “less prudent” burglars, than we might also possibly expect more of their profiles on other items in the apartment. However, we would not see those suspect profiles, because we do not analyze additional samples after having successfully established a profile from window glass.

In the years from 2009 to 2017, between 30 to 40% of all the DNA trace profiles submitted every year to the Swiss DNA database originated from burglary cases [15]. From our study, we would expect that at least 75% of those profiles originate from residents. The number of non-suspect profiles could even be higher, because we cannot be sure that we obtained reference samples from all people authorized to the crime scene, e.g., visitors or first responding local police officers, and DNA might also be transferred and subsequently picked up [3,4,5,6].

In routine casework, we usually do not obtain reference samples from inhabitants. From the 144 samples in the present study, we normally would have sent 44 unique profiles into CODIS (redundant profiles from the same case would not be submitted and were subtracted). After the comparison with the reference samples, we ended up submitting 11 profiles (10 single/major component profiles and 1 mixture). To date (10 December 2021), six of those profiles generated hits with profiles from persons registered in the Swiss DNA database, five of which originated from samples taken from window glass, underlining the high potential of this trace type to advance burglary investigations.

To date, the Swiss DNA database contains around 100,000 trace DNA profiles. Therefore, extrapolating the results from of our study, we expect that around 25,000 trace DNA profiles registered in the database actually originate from inhabitants. Not being excluded from a trace DNA profile as a resident bears the risk of being associated with other offences by a trace-to-trace match in CODIS, as mentioned in the introduction. However, a lack of prior exclusion interferes also with the privacy of the residents in a more general way. Today, the standard STR profiles contain only little personal information. However, potentially more privacy intrusive techniques, such as forensic DNA phenotyping (FDP) are on the rise [16]. Even though we consider such a development as not very probable, privacy issues for crime scene authorized individuals might increase in situations where FDP could also be employed for volume crimes in the future.

## 5. Conclusions

More than 70% of all profiles generated from window glass in the vicinity of the break-in site were caused by unknown individuals, whereas less than 10% of all other traces led to unknown, and therefore possible suspect profiles. Therefore, traces from (broken) windows should be prioritized in crime scene sampling for the generation of investigative leads. To minimize the inclusion of DNA profiles originating from crime scene authorized individuals in CODIS, the collection of reference samples should at least be considered for traces that were not sampled from window glass.

## Data Availability

DNA quantification data are stored at the University of Bern and may be made available upon request. No access to DNA profiles will be provided to anyone.
